# Genomic Regions Associated with Drought Tolerance and Other Traits in Lentils (*Lens* sp.)

**DOI:** 10.3390/plants15050674

**Published:** 2026-02-24

**Authors:** Andrea Fernandez-Gutierrez, Alvaro F. Rodriguez-Torres, Shaun Curtin, Ana I. González, Carlos Polanco, Juan J. Gutierrez-Gonzalez

**Affiliations:** 1 Area de Genética, Departamento de Biología Molecular, Universidad de León, Campus de Vegazana s/n, 24071 León, Spain; arodt@unileon.es (A.F.R.-T.); aigonc@unileon.es (A.I.G.); carlos.polanco@unileon.es (C.P.); 2 United States Department of Agriculture, Plant Science Research Unit, Saint Paul, MN 55108, USA; 3 Department of Agronomy and Plant Genetics, University of Minnesota, Saint Paul, MN 55108, USA; 4 Center for Plant Precision Genomics, University of Minnesota, Saint Paul, MN 55108, USA; 5 Center for Genome Engineering, University of Minnesota, Saint Paul, MN 55108, USA; 6 Instituto de Biología Molecular, Genómica y Proteómica (INBIOMIC), Universidad de León, Campus de Vegazana, 24071 León, Spain

**Keywords:** abiotic stress, drought, legumes, lentils, QTL mapping, SNP GBS markers

## Abstract

Drought is one of the major constraints to lentil production worldwide, making the development of drought-tolerant varieties essential for stable yields. Identifying genes and markers linked to drought tolerance is a crucial first step. We analyzed 90 recombinant inbred lines (RILs) derived from an interspecific cross between the drought-susceptible *Lens culinaris* cv. Alpo and the tolerant *L. odemensis* ILWL235 to investigate genomic regions associated with drought tolerance. Using 4163 high-quality SNP markers obtained through Genotyping-by-Sequencing (GBS), we constructed a linkage map showing seven groups corresponding to the lentil chromosomes. The map spans 786.82 cM and covers 3.46 G bp, representing approximately 88% of the lentil genome. To assess drought tolerance, RILs were subjected to water stress under greenhouse conditions by maintaining the soil moisture at a 40% field capacity (FC) in pots for 15 days, with the leaf relative water content (RWC) recorded every two days. Plants were phenotyped for yield, 100-seed weight, and seed number under both control and stress conditions. We identified 26 Quantitative Trait Loci (QTLs) strongly associated with drought tolerance traits and found putative candidate genes for most of them. Additional traits, including stem pigmentation, flower coloration, seed coat patterning, and seed ground color, were also mapped, and their genomic locations validated the accuracy of our linkage map.

## 1. Introduction

Lentils are among the most consumed legumes worldwide, ranking fifth in global production [1]. In developing countries, they are considered one of the most affordable sources of proteins [2]. However, lentil production is increasingly threatened by drought [3]. Water deficits during critical stages such as flowering or pod formation can drastically reduce both yield and seed quality [3,4]. Breeding drought-tolerant varieties offers a promising solution [5], with the identification of genes and markers associated with tolerance as a necessary first step.

Drought tolerance is a complex trait involving adaptations that enable plants to escape, avoid, or endure periods of water scarcity [6]. To avoid desiccation, plants activate mechanisms to regulate the water status, but these strategies are only effective in the short term. Long-term survival requires additional mechanisms, such as radical oxygen species (ROS) scavenging, and osmotic adjustment. Regardless of the strategy, maintaining the internal water balance is essential. Relative water content (RWC) is widely used to assess tissue hydration and serves as a reliable indicator of drought stress. Susceptible genotypes struggle to retain water, leading to desiccation, while tolerant individuals can sustain RWC levels comparable to those of irrigated plants. Leaf RWC has proven to be the most reliable physiological indicator of drought tolerance in lentils under greenhouse conditions [5].

Although lentils exhibit moderate drought tolerance, responses vary among accessions and are largely determined by genetic factors. Wild accessions often harbor a higher frequency of beneficial alleles for stress-related traits compared to cultivated varieties, making them valuable sources for introgression in breeding programs [7]. Stress responses are regulated by signaling pathways that coordinate morphological, physiological, and biochemical adaptations [8]; yet, the specific mechanisms underlying tolerance in individual accessions remain poorly understood. Finding causal genes or linked makers is a crucial step toward uncovering these mechanisms. Approaches such as Quantitative Trait Loci (QTL) mapping and Genome-Wide Association Studies (GWASs) are commonly used to pinpoint genomic regions associated with drought tolerance.

QTL mapping involves generating a segregating population by crossing two parental lines with contrasting values for those traits and determining the association between individual lines and genetic markers. A linkage map determines the order and distances between genetic markers along linkage groups, providing the necessary framework to correlate markers with phenotypic variations. In lentil research, linkage analyses and QTL mapping have been used to find genomic regions associated with important agronomic traits [9] such as plant height, shoot number or pod dehiscence [10], yield [11], flowering time [12], earliness [13], or tolerance to toxic compounds like aluminum [14]. Furthermore, the genomic regions controlling the seed nutrient content have also been identified, for example, for manganese uptake [15].

QTL mapping has facilitated the identification of markers linked to resistance against major lentil biotic stresses, such as Ascochyta blight [16] or anthracnose [17], as well as abiotic stresses, such as drought. Several loci were found associated with drought tolerance at both the seedling and adult stages [4,18]. In seedlings, a key region (LOD score of 19.9) designated *Sdt* was reported in a linkage group later aligned to chromosome 1 (chr-1) [4]. However, the lack of precise localization prevented candidate gene identification. At the adult stage, 14 root and shoot traits contributing to improved performance under a water deficit were found to be tightly associated with up to 18 markers (LOD scores ranging from 2.75 to 8.14) [18]. However, once again, the resolution was insufficient for candidate gene identification. 

GWA studies also aim to identify meaningful marker–trait associations but rely on non-structured natural populations. In lentils, GWASs have identified loci related to root rot resistance [19]. More recently, Kumar et al. [20] analyzed a lentil population of 243 accessions from diverse origins to identify loci linked to several morphological traits in seedlings growing under drought stress. They found 71 SNPs (LOD score > 4) associated with traits such as shoot fresh weight, root fresh weight, or root volume. Interestingly, some of these markers were located near candidate genes involved in detoxification, signaling, and other protective functions.

Various loci linked to drought tolerance have been identified in other legumes, either through QTL or GWAS analyses. For example, in common beans, a few markers in chromosomes 1, 3, and 6 were associated with seed yield and pod weight, both in a biparental population [21] and in a Mesoamerican diversity panel [22]. In another study, as many as 77 SNPs were found linked to drought tolerance through a Genome-Environment Association analysis in common beans [23]. In soybeans, 52 drought-related SNPs were identified through GWASs [24]. Drought-tolerance QTL mapping has also been carried out for non-legume crops, such as sorghum, finding more than 10 linked markers [25], and in rice, where nine regions were identified in a population of RILs [26].

Thus far, studies seeking to unravel the genetic basis of drought tolerance on lentils are limited and only a small number of candidate genes have been proposed. In this study, we used a RIL population developed from an interspecies cross between *L. culinaris* cv. Alpo and *L. odemensis* ILWL235, and thousands of GBS-derived SNP markers to construct a linkage map on which to carry out a drought-stress QTL analysis. Drought stress was imposed in pots under greenhouse conditions by maintaining the soil moisture at a 40% field capacity (FC). On the tenth day of the onset of drought stress, the parental lines have previously been shown to have contrasting phenotypes for drought tolerance [5]. Thus, our aims were, first, to identify genomic regions associated with drought-tolerance related traits, and, second, to highlight putative candidate genes that may influence lentils’ tolerance to drought stress.

## 2. Results

### 2.1. Physiological Trait

**Figure 1:**
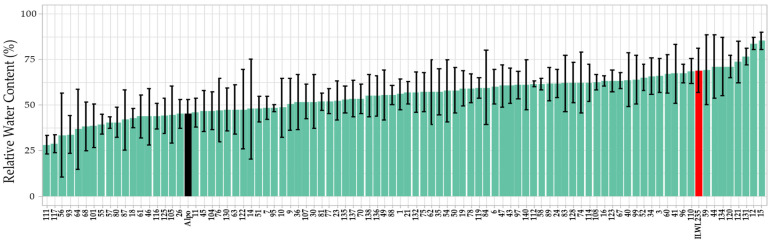
Mean leaf RWC values under drought stress for the RIL population on day 10 after the onset of drought stress. Light-colored bars correspond to the different RI lines, the black bar to *L. culinaris* cv. Alpo parental, and the red bar to *L. odemensis* ILWL235 parental. Whiskers show standard deviations among the 5 replicates.

The average values of leaf RWC were calculated for all control and stressed replicates. On day 10 after the onset of the drought stress, the RWC of the control samples ranged from 58.65% to 100%, while drought-stressed plants had RWC values ranging from 28.08% to 85.25% (Figure 1). The parental lines showed significant differences in RWC between control and stress conditions. The average RWC of *L. culinaris* cv. Alpo was 79.54% under control conditions, and 45.30% under stress conditions (*p*-value = 0.036). On the other hand, *L. odemensis* ILWL235 had a RWC of 82.22% on average under control conditions and 68.92% under stress conditions (*p*-value = 0.057).

In addition, a relative value (ratio) for the leaf RWC was calculated as the percentage of the average RWC of each stressed RIL and parental lines compared to the same lines growing in control conditions. These relative RWC values ranged from 39.05% to 100%, with the parental lines showing values of 56.95% for Alpo, and 83.82% for ILWL235.

### 2.2. Yield Traits

**Figure 2:**
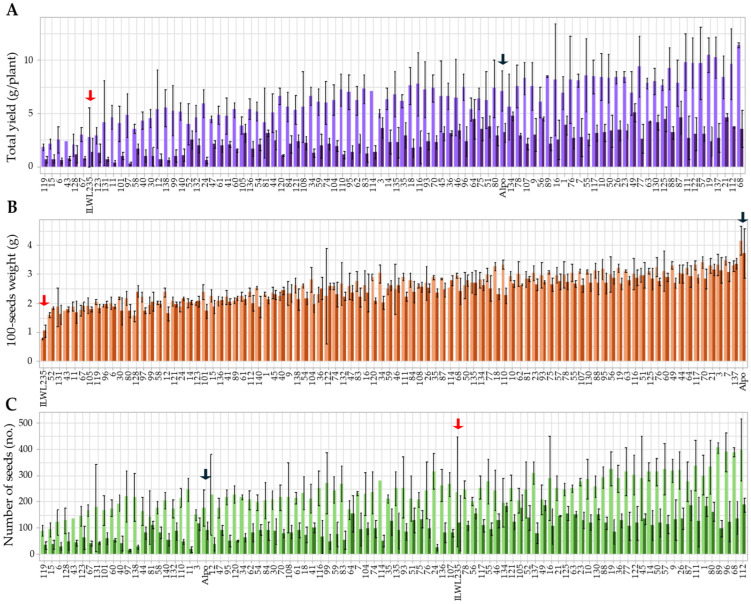
Mean yield trait values for each RIL and the parental lines. (**A**) Total yield represents the average production for each RIL under control (purple) or drought conditions (dark purple). (**B**) 100-seed weight shows the average weight of 100 seeds of each RIL under control (orange) or drought conditions (dark orange). (**C**) Number of seeds represents the average number of seeds produced by each RIL under control (green) or drought conditions (dark green). Whiskers show standard deviations among replicates. Black arrows point at *L. culinaris* cv. Alpo parental and red arrows at *L. odemensis* ILWL235 parental.

Several yield traits, such as the total yield, the number of seeds per plant, and the weight of 100 seeds, were also gathered after the life cycle of the plants ended. For all these traits, seeds produced by each replicate and parental lines were harvested, weighed, counted, and differences between control and stressed individuals calculated (Figure 2).

All yield traits were segregated under both control and stress conditions, with normal distributions confirmed by a Shapiro–Wilks test (*p* > 0.05). Drought stress consistently reduced the total yield, 100-seed weight, and seed number compared with controls. Under drought conditions, the total yield ranged from 0.28 g to 5.07 g, corresponding to losses of 15.67–94.26% relative to controls. For the 100-seed weight, values varied between 1.04 g (ILWL235) and 3.73 g (Alpo). Furthermore, the 100-seed weight values ranged from significant decreases to maintaining values compared to controls. The seed number under drought ranged from 15.67 and 187.67 seeds, which corresponded to 7.82–92.91% fewer seeds produced in the stressed plants than in the controls.

### 2.3. Other Descriptive Traits

We examined several descriptive binary traits that were distinctive between the parental lines and segregated within the Alpo × ILWL235 mapping population (Table 1). These included the tendril type, flower color, stem pigmentation, seed coat pattern, and ground color. The tendril type followed a 3:1 segregation ratio, while others like the seed coat pattern and ground color had a 1:1 segregation ratio (Chi-square test with 1 freedom degree, χ^2^ *p* > 0.05). The flower color and stem pigmentation traits did not fit either a 3:1 or 1:1 segregation ratio (Chi-square test with 1 freedom degree, χ^2^ *p* < 0.05).

### 2.4. High-Density Linkage Map Construction

The 90 RI lines plus the two parents were genotyped using a GBS approach, and a linkage map was constructed using high-quality filtered GBS-SNPs. After the removal of highly heterozygous individuals, and individuals with a high percentage of missing data, 80 RILs were finally used for the linkage map construction. The map has a total of 4163 high-quality markers (Table S1). These markers covered 786.82 cM, distributed in seven linkage groups, corresponding to the seven lentil chromosomes, with an average length per chromosome of 112.40 cM, with chromosome 5 being the shortest (57.39 cM) and chromosome 3 the longest (159.62 cM). The bin interval size varied across the genetic map, with an average of 1.70 cM per bin, and ranging from 1.34 cM in chromosome 1 to 3.03 cM in chromosome 7.

Among the GBS-SNP markers, 39.79% showed segregation distortion, with the distortion distribution being irregular across linkage groups. The highest percent distortion was found in chromosome 7 with 73.68%, and the lowest in chromosome 3 with 2.15% of the markers showing distorted segregation (Figure S1).

### 2.5. QTL Mapping

A multiple interval mapping (MIM) approach was used to detect QTLs. MIM fits several markers and their epistatic interactions simultaneously in a mixed model (see Materials and Methods and Figure S2). The MIM model was iteratively adjusted to identify and refine each QTL position until reaching stability. Finally, epistatic interactions between QTLs were added to the model if statistically significant.

#### 2.5.1. ‘Physiological Traits’ QTLs

The leaf RWC at day 10 was investigated using two related parameters: the net value under drought stress (*rwc*) and the percentage of the RWC under drought stress, each compared with their corresponding control values (*rwc%*).

**Figure 3:**
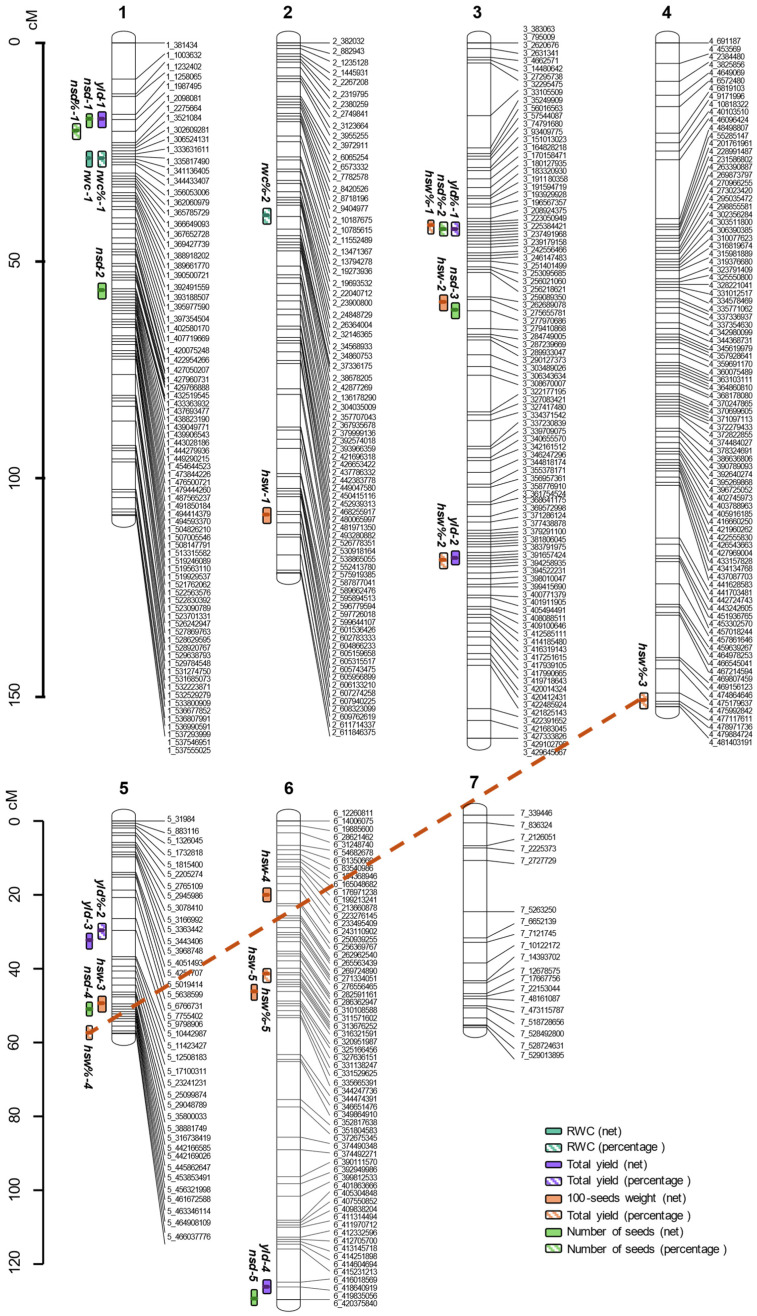
QTL locations for different drought-related traits. Blue ovals indicate ‘leaf RWC’ loci, purple ovals indicate ‘total yield’ loci, orange ovals indicate ‘100-seed weight’ loci, and green ovals indicate ‘number of seeds produced’ loci (legend box). Dark colors correspond to net values and pale ones to percentage values. The name of each QTL is composed of the abbreviation of the trait; “rwc” for RWC on day 10; “yld” for total yield; “hsw” for 100-seeds weight; and “nsd” for number of seeds; followed by the number of QTL for each trait. Orange dotted line represents epistatic interactions.

Regarding the RWC net values under drought stress, a stable model was reached with one major QTL (LOD = 4.45) at position 26.75 cM in chr-1 (*rwc-1*) (Figure 3). This QTL explained 22.87% of the observed variation and had an estimated additive effect of 6.00 (Figure S3).

When using the percentage of RWC of drought-stressed plants relative to controls, a stable model was reached with two QTLs without epistatic interactions (global LOD = 7.15, and 30.65% variance explained). These two QTLs were located at positions 26.75 cM in chr-1 (*rwc%-1*), and 39.94 cM in chr-2 (*rwc%-2*). The individual LODs and estimated additive effects of each QTL were 5.27 and 0.07, respectively, for *rwc%-1*; and 2.60 and 0.08 for *rwc%-2*.

#### 2.5.2. ‘Yield Traits’ QTLs

For yield traits, the following measurements were taken: the mean total net yield of each RIL under control conditions; the mean net yield under drought stress; and the percentage of yield under drought compared with control.

A QTL mapping on net yield values in control conditions retrieved a stable model with four QTLs and no epistatic interactions (global LOD = 14.52, 51.65% variation explained). The QTLs were located at 31.65 cM in chr-2 (*yldC-1*), 51.62 cM in chr-5 (*yldC-2*), and two (48.85 and 64.56 cM) in chr-6 (*yldC-3* and *yldC-4*). The individual additive effects were −0.58, −1.29, 0.76 and −0.87, for *yldC-1* through *yldC-4*, respectively.

For the net yield under drought stress, a stable model was reached with four QTLs without interactions (global LOD = 15.19, 53.27% variance explained). These QTLs were located at positions 17.65, 118.29, 32.50, and 126.31 cM in chr-1 (*yld-1*), chr-3 (*yld-2*), chr-5 (*yld-3*), and chr-6 (*yld-4*), respectively (Figure 3). The estimated additive effects were −0.56 for *yld-1*, −0.27 for *yld-2*, −0.30 for *yld-3,* and −0.34 for *yld-4*.

Finally, the percentage of the total yield of the drought-stressed samples compared to controls reached a stable model with 2 QTLs, at positions 42.87 cM in chr-3 (*yld%-1*), and 29.65 cM in chr-5 (*yld%-2*). The global LOD score of this model was 5.65, with 24.64% of the total variance explained and estimated additive effects of 0.06 for *yld%-1,* and -0.06 for *yld%-2.*

#### 2.5.3. ‘100-Seed Weight’ QTLs

Similarly, we used the 100-seed weight net values under control and drought conditions, as well as the ratio of 100-seed weight of stressed plants versus controls.

First, the 100-seed weight in control samples gave a stable model with five QTLs and two epistatic interactions (global LOD = 26.58; 73.56% variance explained). The QTLs were detected at the following positions: 103.69 cM in chr-2 (*hswC-1*), 37.67 cM in chr-3 (*hswC-2*), 49.57 cM in chr-4 (*hswC-3*), 50.92 cM in chr-5 (*hswC-4*), and 46.21 cM in chr-6 (*hswC-5*). The estimated additive effects were −0.13 for *hswC-1*, −0.06 for *hswC-2*, 0.09 for *hswC-3*, −0.43 for *hswC-4*, and 0.12 for *hswC-5*. Furthermore, epistatic interactions were significant between the QTLs *hswC-2* and *hswC-3* (LOD = 2.50, additive effect = 0.07); and between *hswC-3* and *hswC-5* (LOD = 2.73, additive effect = −0.01).

For the 100-seed weight net values under drought stress, a final stable model (global LOD = 19.51 and 62.34% variance explained) considered five QTLs without interactions. These QTLs were located at the following positions: 108.35 cM in chr-2, 60.00 cM in chr-3 (*hsw-1*), 50.92 cM in chr-5 (*hsw-2*), and 20.00 (*hsw-3*) and 46.21 cM in chr-6 (*hsw-4*). Their estimated effects were −0.11, −0.14, −0.27, −0.17, and 0.19, respectively (Figure 3).

Lastly, the 100-seed weight ratio reached a stable MIM model with five QTLs and one epistatic interaction (global LOD = 15.79, 54.64% of variance explained). The QTLs were at 42.23 cM and 118.92 cM in chr-3 (*hsw%-1* and *hsw%-2*), at 150.99 cM in chr-4 (*hsw%-3*), at 57.40 cM in chr-5 (*hsw%-4*), and at 41.75 cM in chr-6 (*hsw%-5*). The epistatic interaction was between the QTLs *hsw%-3* and *hsw%-4.* The corresponding additive effects were estimated at 0.03, −0.05, 0.05, 0.04, and 0.03 for each QTL, and at 0.03 for the epistatic interaction.

#### 2.5.4. ‘Total Number of Seeds’ QTLs

Similarly, a QTL analysis was also computed for the total number of seeds produced in control and drought conditions, as well as the percentage of seeds produced under drought compared with controls.

With the number of seeds produced in control conditions, a stable MIM model was reached with four QTLs and no epistatic interactions (global LOD = 10.12, 39.76% variance explained). These QTLs were located at positions 30.56 and 42.77 cM in chr-1 (*nsdC-1* and *nsdC-2*), 68.17 cM in chr-3 (*nsdC-3*), and 115.96 cM in chr-6 (*nsdC-4*). Their estimated additive effects were −38.60, 28.80, 22.86, and −19.41, respectively.

For the total number of seeds from plants under water deficits, the stable model was reached with five QTLs without epistatic interactions (global LOD = 16.81, 56.88% variance explained). The QTLs were mapped at positions 17.65 and 57.10 cM in chr-1 (*nsd-1* and *nsd-2*), at 61.53 cM in chr-3 (*nsd-3*), at 50.92 cM in chr-5 (*nsd-4*), and at 129.69 cM in chr-6 (*nsd-5*). The additive effects were estimated to be −15.92, 14.01, 14.62, −16.72, and −10.59 for each QTL, respectively (Figure 3).

Lastly, when we used the relative number of seeds produced in the stressed plants compared to controls, a stable model was reached with two QTLs and no interactions (global LOD = 5.38 and 23.61% variance explained). The QTLs were located at positions 20.28 cM in chr-1 (*nsd%-1*) and at 42.87 cM in chr-3 (*nsd%-2*), with estimated additive effects of −0.08 and 0.05, respectively. The QTL regions detected for all yield traits, in control and drought conditions, are summarized in Table 2 and Table S2, respectively.

#### 2.5.5. QTLs for Other Descriptive/Binary Traits

**Figure 4:**
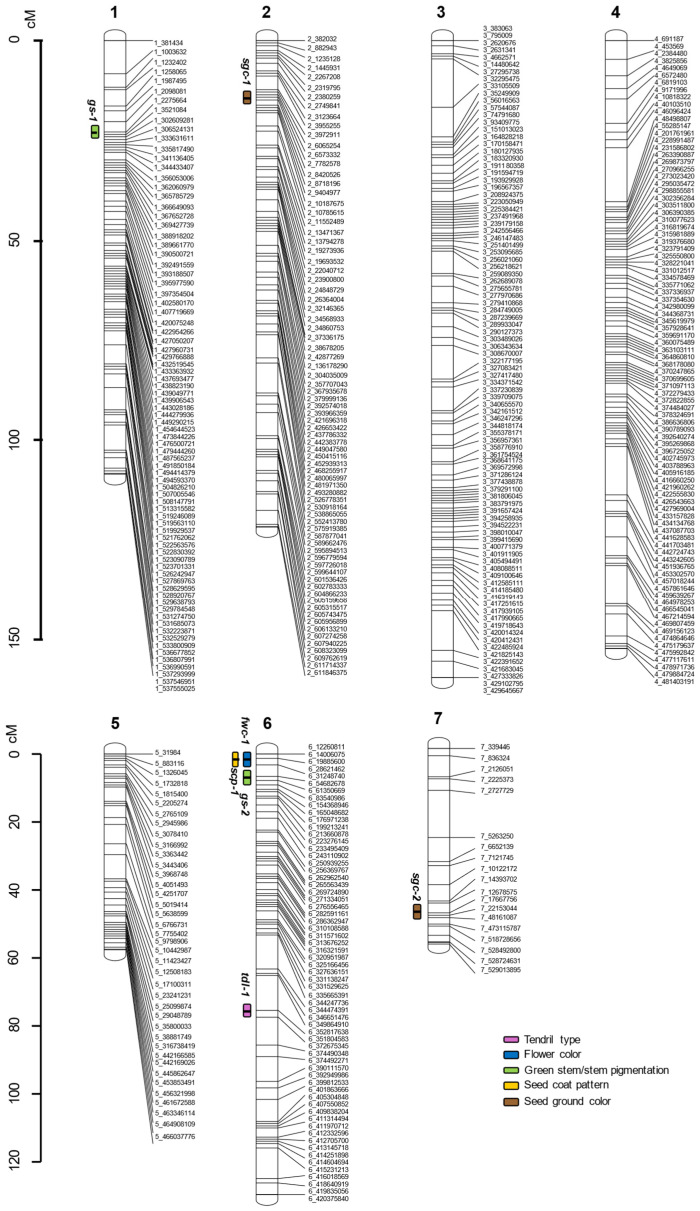
QTL locations were detected for descriptive traits. Pink ovals indicate tendril type loci, blue ovals indicate flower color loci, green ovals indicate stem pigmentation loci, yellow ovals indicate seed pattern loci, and brown ovals indicate seed ground color loci (legend box). The name of each QTL is composed of the abbreviation of the trait: “tdl” for tendril type; “fwc” for flower color; “gs” for stem pigmentation; “scp” for seed coat pattern; and “sgc” for seed ground color; followed by the number of QTL for each trait.

A QTL mapping was also carried out for some descriptive/binary traits, that is, the tendril type, flower color, stem pigmentation, seed coat pattern, and seed ground color (Figure 4 and Table S3).

The type of tendril was associated with a single QTL at position 75.5 cM in chr-6 (*tdl-1*). The stable model reached a global LOD score of 5.46 and explained 27.27% of the variance. The estimated effect for *tdl-1* was −6.15.

The flower color was linked only with one region, at 1.3 cM in chr-6 (*fwc-1*), which showed a LOD score of 4.86, and an estimated effect of 0.75, and explained 17.69% of the variance.

Two QTLs were detected for the stem pigmentation, at positions 22.5 cM of chr-1 (*gs-1*) and 6.6 cM in chr-6 (*gs-2*). These QTLs reached a stable model without interactions, explaining 27.23% of the observed variance, and with a global LOD of 7.94. The estimated effects were −0.87 for *pgm-1* and −0.69 for *pgm-2*.

For the seed coat pattern, position 1.3 cM in chr-6 (*scp-1*) was the only QTL detected, with a LOD score of 21.57, an estimated effect of 8.56, and 71.57% of the variance explained.

Finally, the seed ground color was associated with two genomic regions, at 14.2 cM in chr-2 (*sgc-1*) and at 48.4 cM in chr-7 (*sgc-2*). The final model reached a LOD score of 11.15 and explained 48.22% of the observed variance. The estimated additive effects for *sgc-1* and *sgc-2* were 6.83 and −12.56, respectively.

### 2.6. Candidate Genes for Drought Tolerance

The QTL mapping for drought-related traits highlighted several loci (Table S2 and Table 2). These loci likely influence the drought tolerance, at least to some extent, and deserve deeper scrutiny. Thus, to narrow down the QTL intervals and the number of genes to examine, we considered a 1 cM confidence interval (CI) before and after each QTL with the highest LOD value and converted it into physical positions according to the CDC Redberry Genome Assembly v2.0 [27] (Table 3).

A thorough scrutiny of the confidence intervals returned a number of genes ranging from 31 to 2209, depending on the interval. All genes were further examined in the search for drought-tolerance candidates.

In chr-1, several potential candidate genes were found. For the *nsd-1*/*yld-1* region, *Lcu.2RBY.1g001570* was found near the highest LOD peak, which is described as an outward rectifying potassium channel protein. Within the *nsd%-1* interval is *Lcu.2RBY.1g002260*, a histone-lysine N-methyltransferase ATXR6-like protein, associated with the regulation of DNA expression. Within the *rwc-1/rwc%-1* region, several candidate genes were found (Table S4), including an abscisic acid stress ripening-related protein (*Lcu.2RBY.1g044580*), a GRAS transcription factor (*Lcu.2RBY.1g042390*), two salt tolerance-like proteins (*Lcu.2RBY.1g043620* and *Lcu.2RBY.1g043630*), and a DUF4228 protein (*Lcu.2RBY.1g042860*). Lastly, for *nsd-2*, a calmodulin ortholog of *Lotus japonicus CaM4* is encoded by the *Lcu.2RBY.1g060750* gene, involved in Ca+ transport and sensing in stress signaling mediated by calcium.

In chr-2, within the *rwc%-2* interval, several candidate genes have been annotated, such as four desiccation PCC13-like proteins (*Lcu.2RBY.2g015230*, *Lcu.2RBY.2g015330*, *Lcu.2RBY.2g015340,* and *Lcu.2RBY.2g015350*), two MYB transcription factors (*Lcu.2RBY.2g013990* and *Lcu.2RBY.2g014130*), a heat shock protein (*Lcu.2RBY.2g014400*), or a UDP-D-glucose/UDP-D-galactose 4-epimerase (*Lcu.2RBY.2g019380*), while, within the *hsw-1* interval, a WRKY transcription factor was found (*Lcu.2RBY.2g014360*).

Within the chr-3 confidence intervals, several candidate genes for *hsw%-1*, *hsw-2/nsd-3,* and *yld-2/hsw%-2* were found. Within the *hsw%-1* interval, the *Lcu.2RBY.3g028380* gene was found, an Ulp1 protease family protein, which takes part in the SUMO cycle regulating post-transcriptional changes to stresses like drought. In the region of *hsw-2/nsd-3,* two genes were found that are related to drought tolerance, that is, *Lcu.2RBY.3g042630*, encoding a late embryogenesis abundant (LEA) protein, and *Lcu.2RBY.3g042600*, the protein Ethylene-Insensitive 3 (EIN3) gene. Finally, within the interval of *yld-2* and *hsw%-2*, an EIN3-binding F-box-like protein (*Lcu.2RBY.3g066400*) was found.

For the *hsw%-3* region, *Lcu.2RBY.4g076950* was identified, which encodes for an ortholog of the transcription factor WRKY20. In addition, the *yld%-2/yld-3* region harbors an ortholog of the gene *GmRLK1* (*Lcu.2RBY.5g005340*), which encodes a non-specific serine/threonine protein kinase involved in the ABA pathway.

Finally, in chr-6, confidence intervals for all loci were found to harbor drought-tolerance candidate genes. For the *hsw-4*, an ortholog of the LEA protein SLE1 was found (*Lcu.2RBY.6g029870*). Moreover, the *Lcu.2RBY.6g046660* gene, near the *hsw%-5* locus, encodes a BZIP transcription factor FDL19, and *Lcu.2RBY.6g049240*, within the *hsw-5* interval, is a putative gene for DET1- and DDB1-associated (DDA1) protein. The region of *yld-4* harbors *Lcu.2RBY.6g070510,* a L-fucokinase/GDP-L-fucose pyrophosphorylase. Lastly, for the *nsd-5* locus, two genes were found: *Lcu.2RBY.6g070850* (transcription factor LHY) and *Lcu.2RBY.6g070730* (subtilisin-like serine protease).

## 3. Discussion

Although genome-wide marker discovery has reduced costs and improved accuracy in recent decades, identifying meaningful marker–trait associations in lentils remains challenging due to their large genome. Conversely, QTL analyses are hindered by the lack of high-throughput evenly distributed markers. To address this, we built a genetic map of 4163 high-quality GBS-derived SNP markers, enabling QTL analyses of drought tolerance and other descriptive traits.

In previous endeavors, Topu et al. [28] published a linkage map of 91 RILs based on 458 SSR markers, and Verma et al. [29] built a map with 647 SSR markers, both using intraspecific *L. culinaris* crosses. By using GBS-derived SNPs, with a much higher presence than SSRs, we were able to highlight a higher number of markers compared to those of previous lentil maps. Furthermore, the use of an interspecific cross between cultivated and wild lentils is especially useful for underlining loci associated with disease-resistance traits. In this context, Gela et al. [17] and Adobor et al. [30] published two interspecific lentil linkage maps, the first one with a RIL population derived from *L. culinaris* Eston x *L. ervoides* IG72815, and the second from a cross between *L. culinaris* cv. Eston x *L. ervoides* L01-827A. The authors found two and four loci linked to anthracnosis resistance and Stemphylium blight resistance, respectively. Both maps employed 5000 SNP markers, comparable to our study.

The same interspecific cross between *L. culinaris* cv. Alpo and *L. odemensis* ILWL235 had previously been used to map regions associated with Ascochyta blight resistance [31], using transcriptome-based SNPs and indels. In terms of the number of markers, our map had a slightly lower marker density, at 1.30 cM/marker versus the 1.23 cM/marker shown in the previous Alpo × ILWL235 map. However, our map spans 3.46 G bp of the 3.92 G bp sequenced genome size [27], compared to the 1.78 G bp spanned in this previous map. Both maps employed a reduced number of RILs, which decreases the number of recombination events observed. Nevertheless, the resolution achieved in our Alpo × ILWL235 map enabled the identification of key genomic regions associated with drought tolerance stress and validated QTLs for other descriptive traits.

The segregation distortion found in the Alpo × ILWL235 population is in consonance with previous findings that have also reported a high distortion in populations of RILs [32]. In addition, the segregation distortions seen in interspecific crosses are typically higher than in crosses of the same species [31,32]. The chromosomes with the lowest segregation distortion (chr-3 and chr-6) and the highest (chr-2 and chr-4) agree with the ones described by Polanco et al. [31]. Similarly, we also found that the distortion is more favorable to the *L. culinaris* cv. Alpo alleles, suggesting an involuntary selection of domesticated traits during population development, rather than genotyping or mapping errors. Nevertheless, the segregation distortion shown in Alpo × ILWL235 does not seem to affect the accuracy of the map, as there is almost no alteration in the marker order compared to the physical map. However, it does affect the ratio of the genomic versus physical distance in the regions where a high segregation distortion is found.

Among all the QTLs found, the loci related to RWC are probably the most informative regarding tolerance to drought. In fact, leaf RWC has been proven to reflect a plant’s physiological stress more accurately than any other water stress parameter in lentils and it is highly correlated with yield traits [5]. Maintaining the RWC under water stress has been associated with various physiological responses in legumes. An initial mechanism to sustain the RWC typically involves stomata closure to avoid water loss via transpiration. As the stress progresses, the synthesis of protective molecules such as osmolytes allows the retention of internal water by osmotic pressure regulation. Stomata closure and osmolyte synthesis are modulated by ABA-dependent signaling, which involves MYB, MYC, NAC, and some WRKY transcription factors, as well as members of the Ca^2+^ signaling pathway [8]. Yet, the genes directly linked with the activation of these RWC-upholding responses in lentils are still unknown.

The main locus involved in the modulation of RWC under water stress in the Alpo × ILWL235 map was in chromosome 1 (*rwc-1/rwc%-1*). This genomic region is probably the most relevant in terms of drought tolerance found in our QTL mapping analysis, and, thus, it was meticulously examined. Several promising candidates for drought tolerance were noticed within the CI.

First, the ABA stress ripening (ASR)-related protein is a good candidate to confer enhanced drought tolerance, as the expression of ASR proteins is enhanced under stress conditions, both in an ABA-dependent and ABA-independent manner [33]. In fact, some ASR members have been associated with increased drought tolerance [34]. ASR proteins have been characterized in several plant species such as tomato, wheat, or rice [33].

Second, a GRAS transcription factor was identified in the region. Transcription factors belonging to the GRAS family are involved in several developmental processes as well as in signaling responses under abiotic stresses [35]. Abiotic stresses such as drought and salinity have been shown to induce GRAS expression, triggering the activation of stress response genes [36]. Moreover, the overexpression of some GRAS members has been proven to enhance drought tolerance by reducing ROS accumulation and increasing the antioxidant activity [37].

Third, two salt-tolerance-like proteins are also present in the region. These salt-tolerance-like proteins belong to the B-box zinc finger family (BBX), extensively described in *Arabidopsis* [38]. Members of this protein family are known to be key transcription factors that modulate biological processes including abiotic stress responses [39]. Orthologs of these genes have been found in legumes and are also related with abiotic stress responses [40]. Some BBX members are induced by cold, heat, salt, or drought stress [41], and are thought to act through the ABA pathway [39]. In concordance with these studies, our findings suggest the existence of salt-tolerance protein (STO)/BBX24 orthologs in lentils that regulate the RWC by the activation of stress-response genes, limiting water loss. Notably, an STO/BBX24 has been described as an important player in drought tolerance in tomato [42]. 

Finally, the DUF4228 (domain of unknown function 4228, belonging to an uncharacterized protein family) protein was proposed to influence drought tolerance in lentils [43], and to be involved in abiotic stress responses, including drought in other species [44]. Although the specific function of this group of proteins is still not known, the expression of some DUF4228 members has been linked to drought and salt stress and also appears to be related with ABA synthesis [44]. Thus, this gene could represent a key role in lentil drought responses. We propose a further dissection of the candidate genes found in this region to validate their association with drought tolerance.

Apart from the *rwc-1/rwc%-1* locus, the other region linked to maintaining RWC under drought stress was localized in chr-2 (*rwc%-2*). This region harbors several candidate genes, like various desiccation PCC13-like proteins, whose function is hypothesized to enhance desiccation tolerance by inhibiting enzymatic activity and degradation [45]. Another remarkable gene identified in this region was a heat shock protein. Heat shock proteins are protective molecules synthesized in response to ABA [46], and linked with stress damage alleviation [47].

The total yield is modulated primarily by *yld-1* in our mapping population. It is also located in chr-1, on a genomic region in which an outward rectifying potassium channel protein was also found (*Lcu.2RBY.1g001570*). This type of potassium channel is responsible for carrying K+ across the plasma membrane and to the xylem sap [48]. Under osmotic stress, the increase in K+ efflux through outward rectifying channels is thought to reduce osmotic pressure in the xylem and trigger stomatal closure [49], thus regulating transpiration and water loss. The GORK and SKOR, two outward rectifying potassium channels, have been previously associated with enhanced salt stress [50]. Moreover, under high oxidative stress, the activation of GORK will induce programmed cell death [51]. Our results suggest that these channels may also be involved in drought stress as it entails both osmotic and oxidative stress components. The expression of this gene will likely be more advantageous at the initial stages of drought stress. However, in the long term, stomatal closure will lead to photosynthesis arrest and, ultimately, premature plant death. Thus, an increased expression of the outward rectifying potassium channel would be coupled with a reduction in productivity. We hypothesize that this gene could be upregulated in ILWL235, leading to the decreased yield seen in this parental versus Alpo, and in agreement with the negative additive effect estimated for the *yld-1* QTL.

The weight of 100-seeds is associated with several loci. Among those, the most important were *hsw%-2*, *hsw%-3*, *hsw-3*, *hsw-4,* and *hsw-5*. In the *hsw%-2* region, there is an EIN3-binding F-box-like protein. This protein is necessary for EIN3 degradation, stopping its transcriptional regulation and, hence, terminating the ethylene response [52]. Interestingly, the additive effect of this *hsw%-2* was strongly negative, reflecting the antagonist effect of this protein in the ethylene pathway and, therefore, in the drought tolerance mediated by this hormone. For the *hsw%-3* region, the most interesting candidate gene encodes the transcription factor WRKY20. The WRKY family of transcription factors are involved in the activation of genes in response to several stresses, including drought [53]. A WRKY20 from *Glycine soja* has been shown to enhance drought stress tolerance in different plant species including soybean [54], and alfalfa [55]. The overexpression of WRKY20 in these species acted through the ABA signaling pathway, leading to an enhanced sensitivity to ABA, a more rapid stomatal closure, and an increased cuticle wax deposition, which resulted in decreased water loss. This response did not affect the final yield, sustaining production under drought conditions [54]. Within the *hsw-4* region, a LEA protein was localized, whose importance in drought responses has already been discussed. This LEA is an ortholog of the soybean SLE1 protein, an EM protein. In soybeans, LEA proteins are differentially expressed after a variety of stresses [55]. In *Arabidopsis*, EM1 influences seed maturation and the desiccation process. In rice, the overexpression of OsEM1 plays a central role in drought-tolerance responses [56]. The function of this protein in lentils is unknown; thus, more research is needed to elucidate the specific pathway or pathways it activates.

Finally, for the *hsw-5* locus, a good candidate encodes a putative DDA1 protein that is part of E3 ligase complexes that are responsible for the ubiquitination of proteins leading to proteasomal degradation [57]. The DDA1 proteins have been shown to promote the degradation of ABA receptors, mitigating the signaling of these phytohormone and its associated responses [58]. Mutations that affect the recognition of these ABA receptors by the ubiquitin ligase complexes result in hypersensitive plants with an enhanced drought tolerance [59]. The differences in the recognition of these DDA1 proteins could explain the variation observed in the 100-seed weight seen among the Alpo × ILWL235 lines.

Regarding the number of seeds yielded under drought stress, they showed a strong association with the *nsd%-1* and *nsd-3* loci. For the *nsd%-1* locus, we found an enzyme with a high similarity to ATXR6, a methyltransferase with DNA stabilization and gene-silencing functions. The lack of this enzyme in *Arabidopsis* leads to the activation of transposable elements and the deregulation of reproductive processes [60]. The role of ATXR proteins in the negative regulation of stress responses has been hypothesized, as the loss of ATXR5 and ATXR6 generates *Arabidopsis* plants with a higher tolerance to oxidative stress [61]. Our results support the hypothesis that ATXR proteins act as repressors of stress-responsive genes in lentils. Concerning the *nsd-3* locus, two putative candidate genes were highlighted. The first is a member of the late embryogenesis abundant (LEA) family, which was originally described as influencing seed development [62] and, later, as having a protective role to different stresses [63]. This protective role includes membrane stabilization, the prevention of protein aggregation, and osmotic buffering [64]. Under drought stress, ABA accumulation promotes the expression of LEA proteins, triggering their protective effects [65]. Thus, the expression of this putative lentil LEA gene may aid in counteracting drought’s detrimental effects, allowing for sustaining the production of a similar number of seeds. The second candidate gene localized in the *nsd-3* region encodes an ethylene-insensitive 3 (EIN3) protein. This protein is a transcription factor involved in the ethylene response pathway [66]. Although the exact mechanisms are not yet known, some evidence suggests the role of ethylene in drought stress responses [67]. For instance, EIN3 proteins can activate antioxidant enzymes, increasing ROS scavenging [68]. In fact, in legumes like *Medicago sativa*, EIN3 homologs have been linked with an enhancement in tolerance to several abiotic stresses [69]. Therefore, the presence of an *EIN3* in the *nsd-3* region may also suggest the implication of the ethylene pathway in drought stress responses in lentils.

We mapped other descriptive traits, which also allowed for validating our Alpo × ILWL235 linkage map. For instance, in a previous map using the same interspecific population, Polanco et al. [31] localized the seed coat pattern (*Scp*) in chr-6, overlapping with a genomic region linked to flower color. These findings agree with earlier analyses that mapped the *Scp* locus in the same chromosome [70] and closely related it to a flower color QTL [71]. In our Alpo × ILWL235 map, *scp-1* overlaps with the QTL controlling the flower color trait (*fwc-1*), both in the beginning of chr-6.

The seed ground color has been proposed to be controlled by two main genes, *Ggc* and *Tgc*, which have been mapped to chr-2 and chr-3, respectively [70]. To produce a brown ground color, it is necessary to have dominant alleles for both genes. The tan ground color is produced by a homozygous recessive *Ggc* allele and a dominant *Tgc* allele [72]. Our cross between a brown parental (*L. odemensis* ILWL235), dominant for *Tgc* and *Ggc*, and a tan one (*L. culinaris* cv. Alpo), dominant for *Tgc* and recessive for *Ggc*, only segregates for the *Ggc* locus. Relevantly, our map appears to validate the location of the *Ggc* gene in chr-2 (*sgc-1*). Additionally, we detected a second QTL for the seed ground color that has not been characterized before in lentils and must be further studied, that is, the *sgc-2* locus.

The stem pigmentation, also known as green stem (*Gs*), has been proposed to be controlled by a single gene, purple being the dominant phenotype and green the recessive [73]. A QTL for *Gs* has been previously mapped to chr-1 [16,31]. Here, we identify two QTLs for *Gs*, one in chr-1 (*gs-1*) coinciding with the former described position, and another one in chr-7 (*gs-2*) that had not been yet described. Finally, for tendril development, no previous QTL study has been found. However, several studies have determined the single gene inheritance of this trait [74]. According to our results, the gene controlling the type of tendril developed would be in chr-6, corresponding to the *tdl-1* locus.

In summary, we have accomplished a SNP-based genetic map from an interspecific lentil RIL population with contrasting phenotypes for drought tolerance. The subsequent QTL mapping led to the identification of a total of 19 QTL regions correlated to the RWC and yield traits under drought stress. Most of the QTLs with higher LOD scores had, in their confidence interval, at least one candidate gene likely associated with drought responses. This work represents the first step into the dissection of the molecular basis behind the drought tolerance in lentils. However, more research is needed to validate candidate genes and identify possible mutations responsible for the differences found among lines.

## 4. Materials and Methods

### 4.1. Plant Material and Growing Conditions

The recombinant inbred lines (RILs) of an interspecific cross between *L. culinaris* cv. BGE025606 (Alpo) and *L. odemensis* ILWL235 (Alpo × ILWL235) were used for this study. The cultivated accession Alpo is a selected local variety from breeders in León province (Northern Spain) while ILWL235 is a wild relative originally from Syria and obtained from the ICARDA (Center for Agricultural Research in the Dry Areas) in Syria. The F_7_ Alpo × ILWL235 population comprises 90 RILs.

Seeds were sown in peat moss cups until germination, and seedlings were transferred to pots filled with 550 g of peat moss and wood fiber substrate. Seeds of the wild relative ILWL235 were previously scarified with a soldering iron, avoiding harming the embryo. Plants were grown until maturity in a randomized complete block design (RCBD), with five replicates of stressed plants and three well-watered controls for each accession.

### 4.2. Drought Treatment

When plants reached the onset of the pod formation stage (equivalent to R4 seed maturity stage in soybeans), drought stress was imposed until they attained 40% field capacity (FC), following the protocol first described in Gutierrez-Gonzalez et al. [75], and, later, adapted for lentils [5]. Briefly, pots were weighed daily, and their soil moisture quantified with the Advanced Soil Moisture Sensor TEROS 12 coupled with the ZSC Bluetooth Sensor Interface (METER Group, Washington, DC, USA). When the desired stress levels were reached, the difference in weight between two consecutive days was considered as the amount of water loss for each plant per day and, therefore, the weight of water to be applied to each pot to keep the soil moisture at the stress levels (40% FC). Stressed soil moisture was maintained for 15 days. Control replicates were well-watered to maintain approx. 80% FC moisture.

### 4.3. Relative Water Content

Leaf relative water content (RWC) was determined for control and stressed plants using the formula RWC (%) = (Fresh weight − Dried weight)/(Turgid weight − Dried weight) × 100, and the protocol described by Singh et al. [4]. Briefly, leaf samples were weighted immediately after sampling between 8:00 and 10:00 AM (fresh weight). Leaf samples were hydrated by submerging them in deionized water in a closed tube for 4–6 h (turgid weight). Next, the leaves were oven-dried at 80 °C for 48 h to measure the dry weight. Each sample consisted of several leaflets per plant, with three replicates. Samples were harvested every two days, from day one, starting of the dry period, to the end of the drought treatment on day 15.

### 4.4. Yield and Other Agronomic Traits

Once all the plants were completely dried, after life cycle completion, their seeds were harvested. The following yield traits were quantified for each RIL and the two parental lines: total weight produced (total yield in g), number of seeds produced, and weight of 100 seeds. Yield loss values were obtained, calculating the difference between control and drought conditions for each line and parameter. Several other traits, such as tendril presence, flower color, stem pigmentation, and seed pattern, were also recorded. All these traits were scored as binary traits, with either “0”/”1” values, “0” being the same phenotype as the parental line *L. culinaris* Alpo, and “1” the same as *L. odemensis* ILWL235, as follows: for tendril presence, 0 = developed or 1 = rudimentary; for flower color, 0 = white or 1 = purple; for stem pigmentation (or green stem, *Gs*), 0 = green or 1 = purple; for seed coat pattern (also known as seed pattern color, *Spc*), 0 = absent or 1 = present; and for seed ground color, 0 = tan or 1 = brown.

### 4.5. DNA Extraction

Two seeds of each RIL and the two parental lines were germinated in vitro. After ten days of growing, seedlings were milled in the presence of liquid nitrogen, and their DNA was extracted using the DNeasy Plant (Qiagen, Hilden, Germany) extraction kit following the standard protocol. DNA concentrations were quantified on a Nanodrop ND-1000 (Thermo Fisher Scientific, Waltham, Massachusetts, USA) spectrophotometer and sent for GBS genotyping procedure at the University of Minnesota Genomics Center.

### 4.6. Genotype-by-Sequencing and SNP Calling

The two-enzyme restriction digestion GBS protocol first described in Poland et al. [76] was adapted by the University of Minnesota Genomics Center. Briefly, DNA was digested with the restriction enzymes *Pst*I and *Msp*I. Adaptor sequences were ligated to individual samples and pooled. Libraries were constructed and sequenced using 158 bp paired Illumina technology. From the raw sequence data, reads were demultiplexed according to their Illumina barcodes, assigned to samples, and trimmed the adaptor sequences as described in https://bitbucket.org/jgarbe/gbstrim/ (accessed 10 October 2025). Only reads above 50 bp long were kept. Reads were aligned to the CDC Redberry Genome Assembly v2.0 [27] using the Burrows–Wheeler Aligner (BWA) [77] with the bwa-mem algorithm v0.7.17 with default parameters. The alignment output files in ‘sam’ format were converted to binary versions ‘bam’ with the view algorithm and sorted with sort algorithm within samtools v1.17 [78]. SNP calling was done using freebayes v1.3.6 [79] with the parameters --use-best-n-alleles 4, --min-alternate-count 2, --min-alternate-total 10, --min-coverage 20, and --ploidy 2, and instructed to output genotype qualities. The output vcf file was filtered with the following constraints: a homozygous genotype call was retained if at least two reads supported it; three reads were needed to support a heterozygous call. SNP calls with quality scores below 10, more than 50% missing data, or more than 20% heterozygous calls were discarded at this stage. More stringent filtering was done during map construction. Heterozygote calls were allowed, except in the parents, and used to compute statistics. Individual’s heterozygous calls were set to missing, prior to linkage grouping. The GBS-derived SNP markers were given the following format: “Lcu.2RBY.Chr1_1003854”. The first part corresponded to the genome assembly name, followed by the chromosome and the physical position. In-house bash and perl scripts were developed to perform the preceding tasks.

### 4.7. Linkage Map Construction

MSTMap [80] on the R/ASMa, and the R/qtl packages for R were used for linkage group construction, genetic mapping, and computing of linkage statistics. During map construction, lines that were sequenced at insufficient depth had a high rate of missing or heterozygous observations, or a high number of double crossovers were removed. GBS-derived SNP markers that aligned with the smaller non-pseudomolecules CDC Redberry Genome Assembly v2.0 contigs were removed for subsequent analysis. Initial group assignment was established using a *p*-value of 1E-15, and the maximum likelihood (ML) objective function. The population type was set to RIL7. Recombination frequencies were converted to centiMorgans (cM) using the Kosambi function. Markers with more than 10% missing observations or minor allele frequencies of 0.05, or less, for either parental allele were removed prior to map construction. Default settings were allowed for other parameters. Stringent filtering removed low-quality markers, with excess of missing or heterozygous sites, prior to map construction (see also previous section). Markers with excess double recombination or suspected of being wrongly positioned were also removed during each iteration of linkage map construction.

### 4.8. QTL Mapping

QTL mapping was carried out using the R/qtl package v1.70 [81]. The functions *scanone* and *cim* were first used to search for candidate loci (LOD > 2) genome-wide. *Scanone* computes a genome scan for a single QTL model while *cim* function does a composite interval mapping followed by an interval mapping. For the *cim* scan, four covariates were considered, with a 20 cM window and 1 cM step. The function *scantwo* was also used for detection of epistatic interactions. For all mapping methods, a Haley–Knott regression with 1000 permutations was considered for error calculation. Finally, a MIM was computed using the functions *fitqtl* and *refineqtl* iteratively until a stable model was reached, selecting only the positions with LOD > 2.0. For all the analysis, conditional genotype probabilities were calculated with the *calc.genoprob* function and 1 cM step. The simulation of genotypes was accomplished with the *sim.geno* function with 2.5 cM step, 0.001 probability of error allowed, and 500 simulation replicates. QTL mapping for the binary-recorded traits followed the same procedures, but selecting the “binary” model for MIM.

### 4.9. Statistical Analysis

All data gathered were analyzed using the software R 4.3.0 [82]. Normality was assessed with the Shapiro–Wilk test. For each parameter, statistical differences between control and stressed samples were estimated by Wilcoxon rank sum test with R core functions. Bar graphics were drawn with the package *ggplot2* [83]. Signification was set at *p*-value < 0.05 for all the statistical tests.

## Figures and Tables

**Table 1:** Number of individuals with each phenotype for the descriptive traits in the Alpo × ILWL235 population.

	N.	“0” (=*L. culinaris* cv. Alpo)	“1” (=*L. odemensis* ILWL235)	3:1 χ^2^ (*p*-Value)	1:1 χ^2^ (*p*-Value)
Tendril type	79	64	15	0.2127	0
Flower color	79	28	51	0.0250	0.0097
Stem pigmentation	79	29	50	0.0162	0.0182
Seed coat pattern	79	35	44	0.0001	0.3113
Seed ground color	78	31	47	0.0028	0.0700

**Table 2:** Summary of the QTLs detected in drought conditions for all yield traits.

Name	Chromosome	Position	Trait	LOD	Effect
*nsd-1*	1	17.65	Number of seeds (net)	2.69	−15.92
*yld-1*			Total yield (net)	6.70	−0.56
*nsd%-1*		20.28	Number of seeds (percentage)	3.95	−0.08
*rwc%-1*		26.75	RWC day 10 (percentage)	5.27	0.07
*rwc-1*			RWC day 10 (net)	4.45	6.00
*nsd-2*		57.10	Number of seeds (net)	3.99	14.01
*rwc%-2*	2	39.94	RWC day 10 (percentage)	2.60	0.08
*hsw-1*		108.35	100-seeds weight (net)	3.55	−0.11
*hsw%-1*	3	42.23	100-seeds weight (percentage)	3.35	0.04
*nsd%-2*		42.87	Number of seeds (percentage)	2.17	0.05
*yld%-1*			Total yield (percentage)	3.23	0.06
*hsw-2*		61.53	100-seeds weight (net)	3.83	−0.14
*nsd-3*			Number of seeds (net)	4.92	14.62
*yld-2*		118.29	Total yield (net)	2.36	−0.27
*hsw%-2*			100-seeds weight (percentage)	6.31	−0.05
*hsw%-3*	4	150.99	100-seeds weight (percentage)	5.99	0.05
*yld%-2*	5	29.65	Total yield (percentage)	3.03	−0.06
*yld-3*			Total yield (net)	2.03	−0.30
*hsw-3*		50.92	100-seeds weight (net)	11.93	−0.27
*nsd-4*			Number of seeds (net)	2.20	−16.72
*hsw%-4*		57.39	100-seeds weight (percentage)	4.44	0.04
*hsw-4*	6	20.00	100-seeds weight (net)	5.50	−0.17
*hsw%-5*		41.75	100-seeds weight (percentage)	2.85	0.03
*hsw-5*		46.21	100-seeds weight (net)	9.32	0.19
*yld-4*		126.31	Total yield (net)	3.35	−0.34
*nsd-5*		129.69	Number of seeds (net)	2.99	−10.59
*hsw%-3*/*hsw%-4*	4–5	-	100-seeds weight (percentage)	2.21	0.03

**Table 3:** Genetic and physical positions of the closest markers, start, end, and number of genes found within the confidence interval for each QTL.

Name	Chr.	Genetic Position (cM)	Closest Marker	Physical Position (pb)	Start Confidence Interval (pb)	End Confidence Interval (pb)	No. Genes
*nsd-1*, *yld-1*	1	17.65	1_2098081	2,098,081	1,987,495	2,275,664	35
*nsd%-1*		20.28	1_2275664	2,275,664	2,098,081	3,521,084	69
*rwc-1,* *rwc%-1*		26.75	1_344433407	344,433,407	335,817,490	356,053,006	340
*nsd-2*		57.10	1_452486146	452,486,146	449,290,215	473,844,226	359
*rwc%-2*	2	39.94	2_42877269	42,877,269	38,678,205	136,178,290	1501
*hsw-1*		108.35	2_606133210	606,133,210	605,956,899	607,274,258	31
*hsw%-1*	3	42.23	3_193928928	193,928,928	191,594,719	208,924,375	201
*nsd%-2,* *yld%-1*		42.87	3_196567357	196,567,357	191,594,719	208,924,375	201
*hsw-2,* *nsd-3*		61.53	3_279410868	279,410,868	277,970,686	284,749,005	147
*yld-2, hsw%-2*		118.29	3_394522231	394,522,231	394,258,935	399,415,690	133
*hsw%-3*	4	150.99	4_478971736	478,971,736	479,884,724	477,117,611	110
*yld%-2,* *yld-3*	5	29.65	5_7755402	7,755,402	6,766,731	9,798,906	128
*hsw-3, nsd-4*		50.92	5_442166585	442,166,585	316,738,419	445,862,647	2209
*hsw%-4*		57.39	5_466037776	466,037,776	464,908,109	474,935,647	437
*hsw-4*	6	20.00	6_223276145	223,276,145	213,660,878	223,276,145	170
*hsw%-5*		41.75	6_320951987	320,951,987	316,321,591	325,166,456	203
*hsw-5*		46.21	6_335665391	335,665,391	331,529,625	344,247,736	202
*yld-4*		126.31	6_419835056	419,835,056	418,640,919	420,375,840	79
*nsd-5*		129.69	6_420375840	420,375,840	419,835,056	420,574,053	37

## Data Availability

All data used in the preparation of the manuscript will be available upon request.

## Supplementary Materials

The following supporting information can be downloaded at: https://www.mdpi.com/article/10.3390/plants15050674/s1, Table S1: Linkage map parameters in each chromosome; Figure S1: Segregation distortion across the Alpo × ILWL235 linkage map. Red bars indicate distortion favorable to *L. culinaris* cv. Alpo alleles, and blue bars indicate distortion towards the *L. odemensis* ILWL235 alleles; Figure S2: Composite Interval Mapping results for the different traits in the RIL population. (A) Relative water content after 10 days of drought conditions; RWC net values are represented in dark blue lines and percentage of RWC under drought comparing with control conditions are in light blue lines. (B) Total yield produced under drought conditions; net values are represented in dark purple lines and percentage of yield produced under drought compared with control conditions is in light purple lines. (C) 100-seed weight produced under drought conditions; net values are represented in dark orange lines and percentage of yield produced under drought compared with control conditions is in light orange lines. (D) Number of seeds produced under drought conditions; net values are represented in dark green lines and percentage of yield produced under drought compared with control conditions is in light green lines. Black lines stand for control values for each trait; Figure S3: Effect plot for different alleles of peak markers at every QTL region detected with MIM mapping. AA genotype represents homozygosis for the allele coming from L. culinaris cv. Alpo parental, whereas BB indicates homozygosis for L. odemensis ILWL235 allele; Table S2: Summary of QTLs highlighted in control conditions; Table S3: Summary of QTLs detected for descriptive traits; Table S4; Summary of drought-related candidate genes found within the rwc-1/rwc%-1 confidence interval.

## Abbreviations

The following abbreviations are used in this manuscript:

BWA	Burrows–Wheeler Aligner
FC	field capacity
GBS	Genotyping-by-Sequencing
GWAS	Genome-Wide Association Studies
MIM	multiple interval mapping
ML	maximum likelihood
QTLs	Quantitative Trait Loci
RCBD	randomized complete block design
RIL	recombinant inbred lines
ROS	radical oxygen species
RWC	relative water content
