# Supplementary material for: Genomic Regions Associated with Drought Tolerance and Other Traits in Lentils (Lens sp.)
Source: Plants (Basel). 2026 Feb 24;15(5):674. doi: 10.3390/plants15050674 (PMC12987374; doi:10.3390/plants15050674)
Supplement: Supplementary file 1 [file plants-15-00674-s001.zip › plants-4141916-supplementary.pdf]

Supplementary material

Table S1. Linkage map parameters in each chromosome.

	Number of SNPs	Length (cM)	Segregation distortion (%)
Chromosome 1	843	108.41	30.86
Chromosome 2	514	121.90	51.95
Chromosome 3	487	159.62	2.15
Chromosome 4	706	152.24	62.22
Chromosome 5	640	57.40	51.28
Chromosome 6	208	129.69	6.35
Chromosome 7	765	57.56	73.68
Total	4163	786.82	39.79

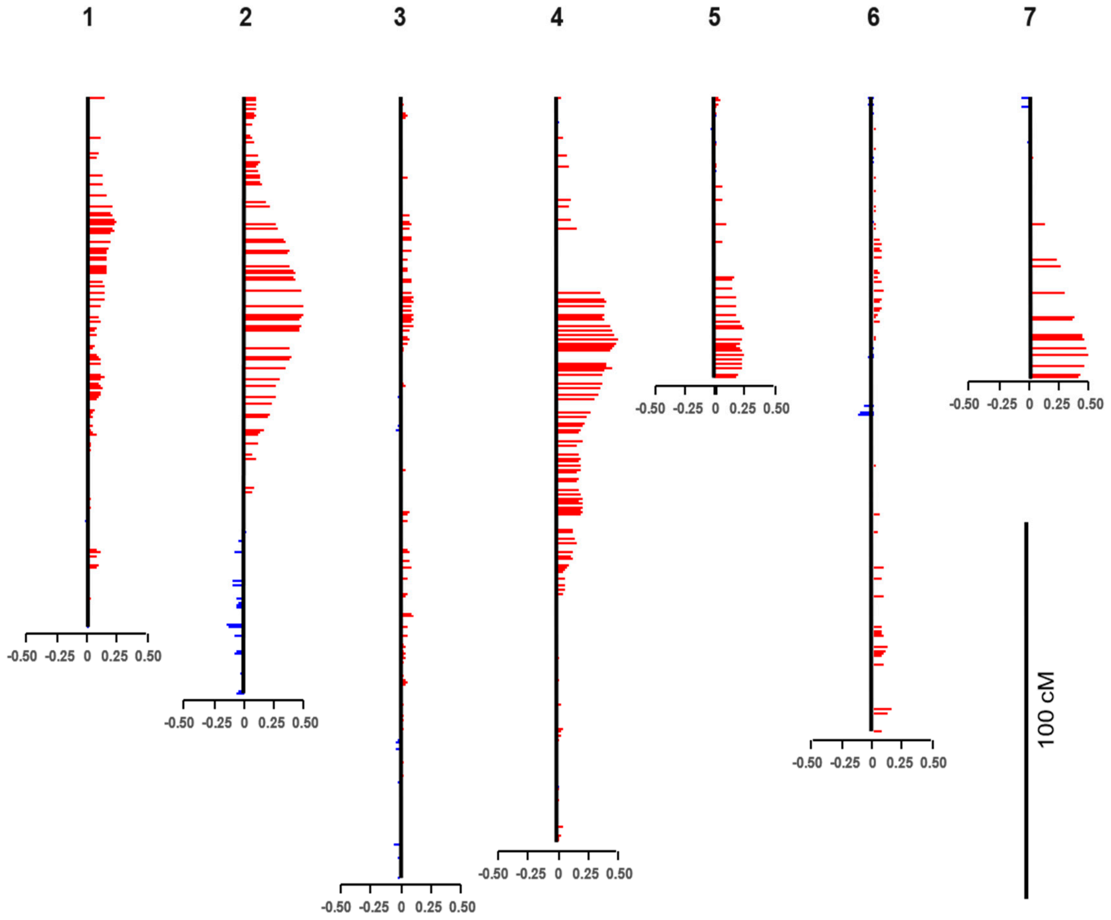

Figure S1. Segregation distortion across the Alpo × ILWL235 linkage map. Red bars indicate distortion favorable to *L. culinaris* cv. Alpo alleles, and blue bars indicate distortion towards the *L. odemensis* ILWL235 alleles.

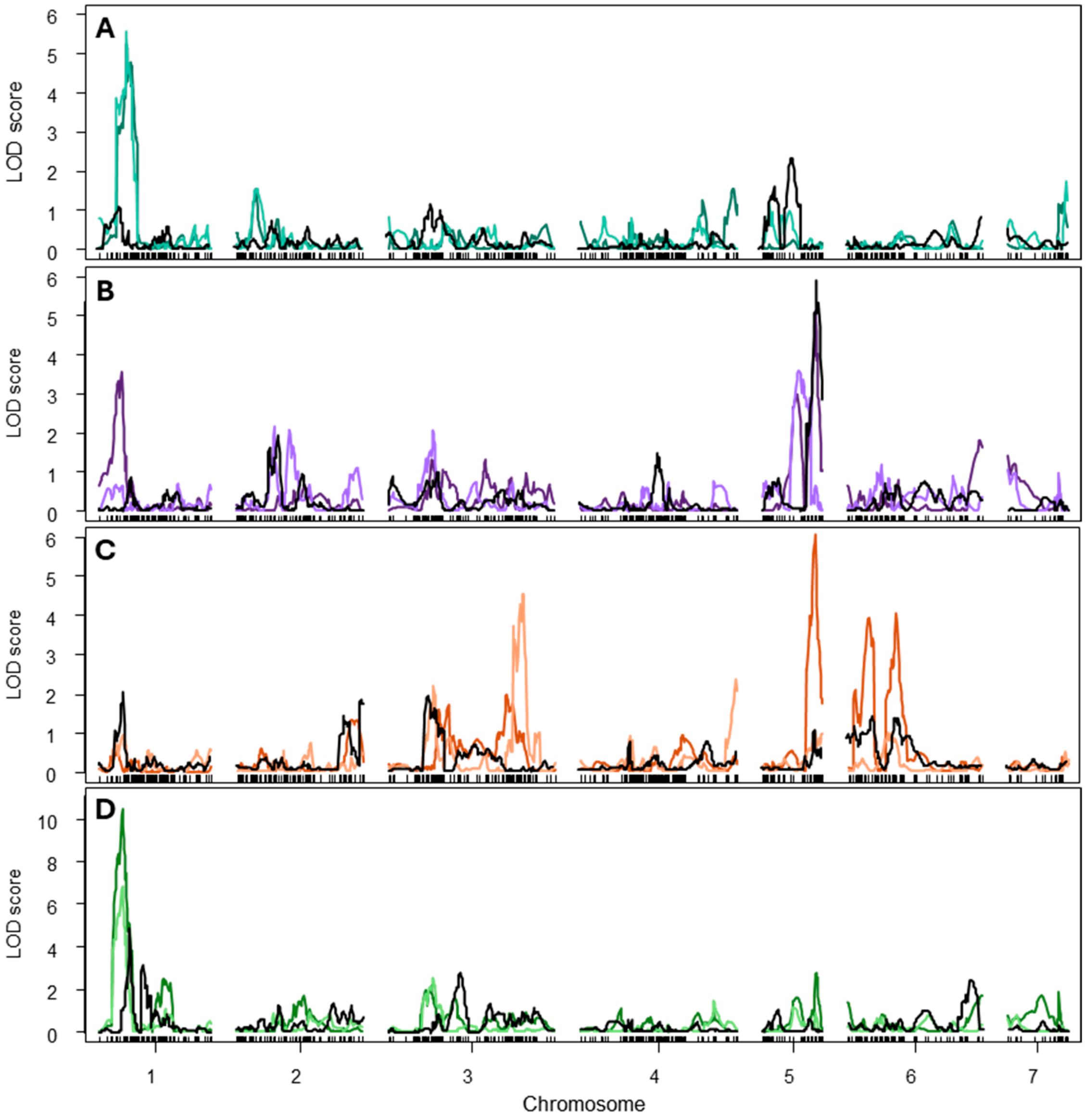

Figure S2. Composite Interval Mapping results for the different traits in the RIL population. A) Relative water content after 10 days of drought conditions; RWC net values are represented in dark blue lines and percentage of RWC under drought comparing with control conditions are in light blue lines. B) Total yield produced under drought conditions. Net values are represented in dark purple lines and percentage of yield produced under drought compared with control conditions is in light purple lines. C) 100-seed weight produced under drought conditions. Net values are represented in dark orange lines and percentage of yield produced under drought compared with control conditions is in light orange lines. D) Number of seeds produced under drought conditions; net values are represented in dark green lines and percentage of yield produced under drought compared with control conditions is in light green lines. Black lines stand for control values for each trait.

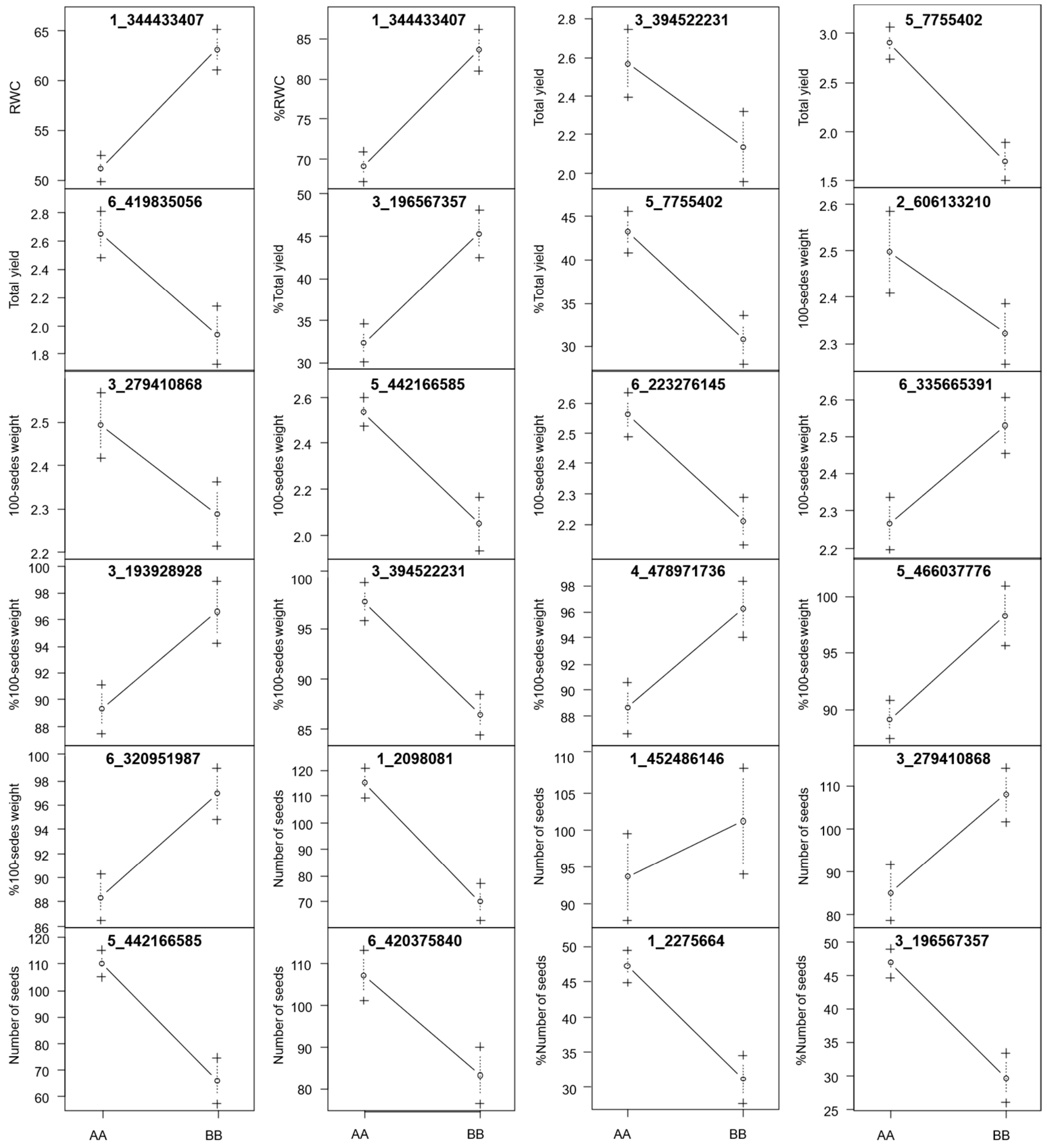

Figure S3. Effect plot for different alleles of peak markers at every QTL region detected with MIM mapping. AA genotype represents homozygosis for the allele coming from L. culinaris cv. Alpo parental, whereas BB indicates homozygosis for L. odemensis ILWL235 allele.

Table S2. Summary of QTLs highlighted in control conditions.

Name	Chromosome	Position	Trait	LOD	Effect
nsdC-1	1	30.56	Number of seeds control (net)	6.4	-38.6
nsdC-2		42.77	Number of seeds control (net)	3.91	28.8
yldC-1	2	31.65	Yield control (net)	2.27	-0.58
hswC-1		103.69	100-seeds weight control (net)	5.24	-0.13
hswC-2	3	37.67	100-seeds weight control (net)	7.06	-0.06
nsdC-3		68.17	Number of seeds control (net)	3.67	22.86
hswC-3	4	49.57	100-seeds weight control (net)	8.76	0.09
hswC-4	5	50.92	100-seeds weight control (net)	23.24	-0.43
yldC-2		51.62	Yield control (net)	10.27	-1.3
hswC-5	6	46.21	100-seeds weight control (net)	6.12	0.12
yldC-3		48.85	Yield control (net)	3.92	0.76
yldC-4		64.56	Yield control (net)	5.04	-0.87
nsdC-4		115.96	Number of seeds control (net)	2.69	-19.41
hswC-2/ hswC-3	3-4	-	100-seeds weight control (net)	2.50	0.07
hswC-3/ hswC-5	4-6	-	100-seeds weight control (net)	2.73	-0.01

Table S3. Summary of QTLs detected for descriptive traits.

Name	Chromosome	Position	Trait	LOD	Effect
gs-1	1	22.50	Stem pigmentation/Green stem	5.55	-0.87
sgc-1	2	14.20	Seed ground color	9.03	6.83
fwc-1	6	1.30	Flower color	4.86	0.75
gs-2	6	6.60	Stem pigmentation/Green stem	2.79	-0.69
scp-1	6	1.30	Seed color pattern	21.57	8.56
tdl-1	6	75.50	Tendril type	5.46	-6.15
sgc-2	7	48.40	Seed ground color	2.68	-12.56

Table S4. Summary of drought-related candidate genes found within the rwc-1/rwc%-1 confidence interval.

Description	Gene ID
Transporters	
ABC transporter-like	Lcu.2RBY.1g043450, Lcu.2RBY.1g043500
Auxin efflux carrier	Lcu.2RBY.1g044640
Auxin influx transporter	Lcu.2RBY.1g043590
Major intrinsic protein transporter	Lcu.2RBY.1g044900
SPX MFS transporter	Lcu.2RBY.1g042730
Sugar porter MFS transporter	Lcu.2RBY.1g043120
Transcription factors	
Abscisic acid stress ripening-related	Lcu.2RBY.1g044580
AP2 transcription factor	Lcu.2RBY.1g044780
GRAS transcription factor	Lcu.2RBY.1g042390
NAC transcription factor	Lcu.2RBY.1g042170, Lcu.2RBY.1g042180
Salt tolerance-like protein	Lcu.2RBY.1g043620, Lcu.2RBY.1g043630
Tubby-like F-box	Lcu.2RBY.1g044530
Receptors	
EF hand calcium-binding	Lcu.2RBY.1g043480
Lectin receptor kinase	Lcu.2RBY.1g043520
LRR receptor-like kinase	Lcu.2RBY.1g041630, Lcu.2RBY.1g041640, Lcu.2RBY.1g041660, Lcu.2RBY.1g041700, Lcu.2RBY.1g041710, Lcu.2RBY.1g041720, Lcu.2RBY.1g043580, Lcu.2RBY.1g043640, Lcu.2RBY.1g043660, Lcu.2RBY.1g043720, Lcu.2RBY.1g043730
Drought-response molecules	
Calmodulin-binding heat-shock protein	Lcu.2RBY.1g042070
CDPK protein	Lcu.2RBY.1g044720
Hsp20	Lcu.2RBY.1g044550
Peroxidase	Lcu.2RBY.1g044820
TCP-1/cpn60 chaperonin	Lcu.2RBY.1g044120
Unknown function	
DUF4228 protein	Lcu.2RBY.1g042860
